# Supplementary material for: Identification of inulin-responsive bacteria in the gut microbiota via multi-modal activity-based sorting
Source: Nat Commun. 2023 Dec 14;14:8210. doi: 10.1038/s41467-023-43448-z (PMC10721620; doi:10.1038/s41467-023-43448-z)
Supplement: Supplementary file 1 — Supplementary Information [file 41467_2023_43448_MOESM1_ESM.pdf]

Supplementary information

Identification of inulin-responsive bacteria in the gut microbiota via multi-modal activity-based sorting

Alessandra Riva[1#], Hamid Rasoulimehrabani[1], José Manuel Cruz-Rubio[2], Stephanie L. Schnorr[1], Cornelia von Baeckmann[3], Deniz Inan[1], Georgi Nikolov[1], Craig W. Herbold[1], Bela Hausmann[4,5], Petra Pjevac[1,4], Arno Schintlmeister[1], Andreas Spittler[6], Márton Palatinszky[1], Aida Kadunic[1], Norbert Hieger[1], Giorgia Del Favero[7], Martin von Bergen[8], Nico Jehmlich[8], Margarete Watzka[9], Kang Soo Lee[10], Julia Wiesenbauer[9], Sanaz Khadem[1], Helmut Viernstein[2], Roman Stocker[10], Michael Wagner[1,11], Christina Kaiser[9], Andreas Richter[9], Freddy Kleitz[3] and David Berry[1,4]

[1]Centre for Microbiology and Environmental Systems Science, Department of Microbiology and Ecosystem Science, Division of Microbial Ecology, University of Vienna, Vienna, Austria.

[2]Department of Pharmaceutical Technology and Biopharmaceutics, University of Vienna, Vienna, Austria.

[3]Department of Functional Materials and Catalysis, Faculty of Chemistry, University of Vienna, Vienna, Austria.

[4]Joint Microbiome Facility of the Medical University of Vienna and the University of Vienna, Vienna, Austria.

[5]Department of Laboratory Medicine, Medical University of Vienna, Vienna, Austria.

[6]Core Facility Flow Cytometry and Surgical Research Laboratories, Medical University of Vienna, Vienna, Austria.

[7]Department of Food Chemistry and Toxicology, Faculty of Chemistry, University of Vienna, Vienna, Austria.

[8]Helmholtz Centre for Environmental Research, Department of Molecular Systems Biology, Leipzig, Germany.

[9]Centre for Microbiology and Environmental Systems Science, Department of Microbiology and Ecosystem Science, Division of Terrestrial Ecosystem Research, University of Vienna, Vienna, Austria.

[10]Institute for Environmental Engineering, Department of Civil, Environmental and Geomatic Engineering, ETH Zurich, Zurich, Switzerland.

[11]Center for Microbial Communities, Department of Chemistry and Bioscience, Aalborg University, Aalborg, Denmark.

Supplementary Figures

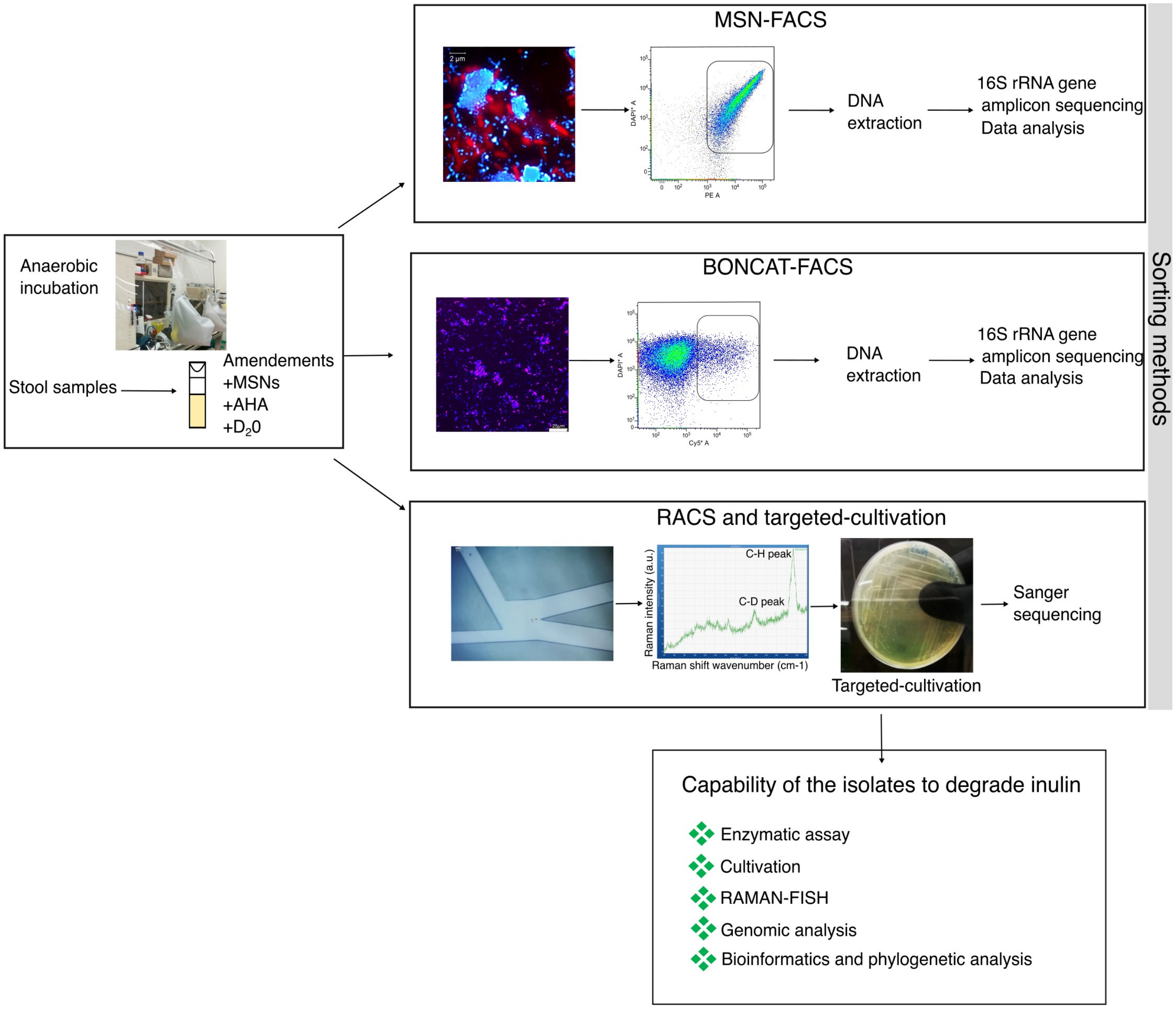

Supplementary Figure 1. Experimental design of the study.

Fresh stool samples were incubated under anaerobic conditions and in the presence of the mesoporous silica nanoparticles (MSNs) functionalized with inulin and labeled with the fluorescence dye rhodamine to evaluate the spatial interaction between gut bacteria and MSNs grafted with inulin. The MSNs together with the bacteria were sorted by FACS and their 16S rRNA genes were amplified and sequenced to detect bacterial taxa able to interact with MSNs. Subsequently fresh stool samples were incubated under anaerobic condition and in the presence of the activity markers [(L-Azidohomoalanine [AHA] and heavy water [D_2O]). Active cells were sorted with BONCAT-FACS

and their 16S rRNA genes were amplified and sequenced. Responder strains were directly isolated and cultivated with RACS and subsequently identified by Sanger sequencing. Further experiments were carried out to study the capability of the isolates to degrade inulin. Fructan assay and cultivation were performed in inulin supplemented medium. RAMAN-FISH was carried out to verify the deuterium incorporation for some *Coriobacteriia* members. Whole genome sequencing, bioinformatics, and phylogenetic analysis were performed for *Coriobacteriia* members *Eggerthella lenta* and *Gordonibacter urolithinfaciens.*

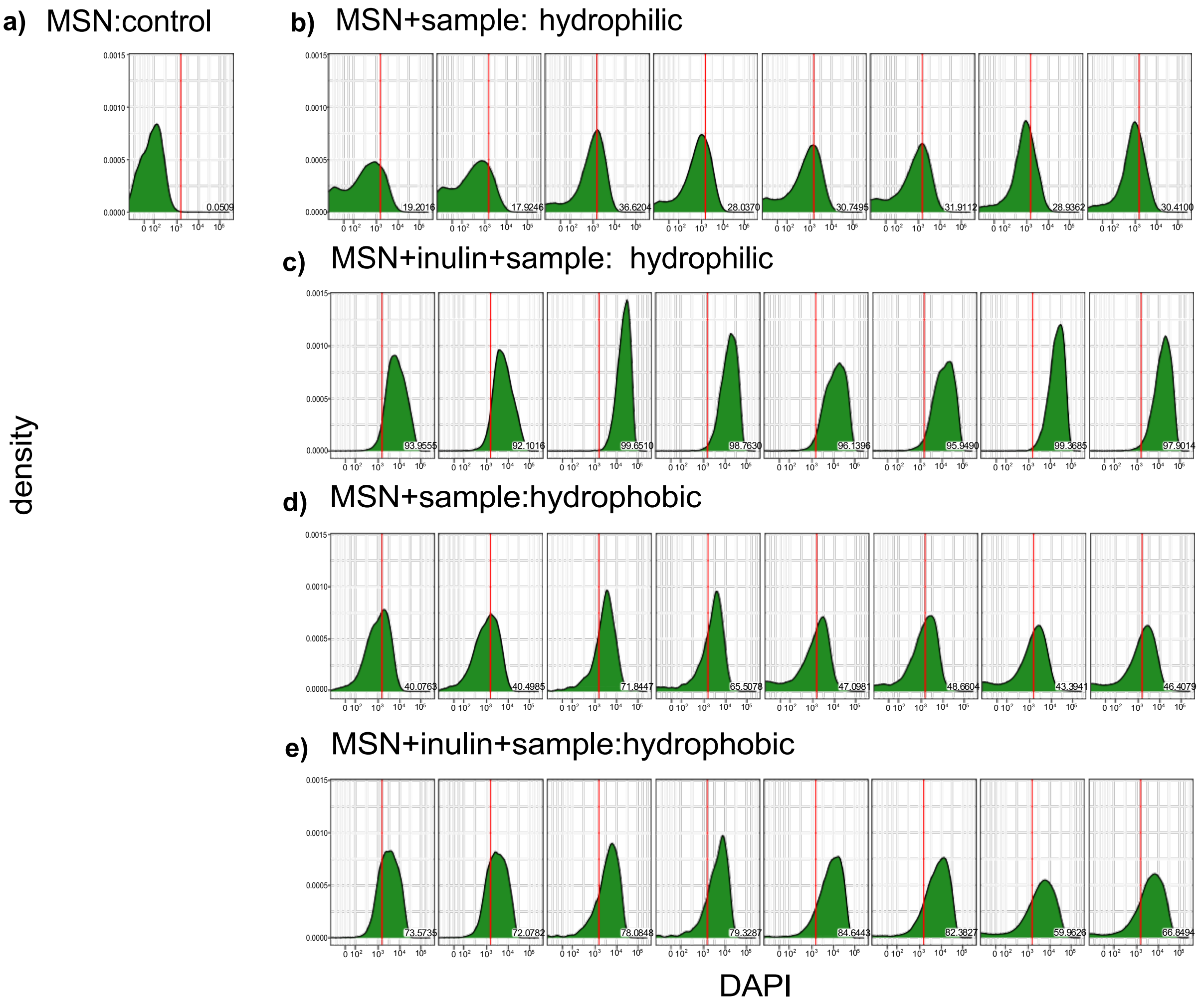

Supplementary Figure 2. Detection of binding bacteria to MSNs after anaerobic incubation via FACS analyses.

Density plots show the intensity of the DAPI signal in the gate encompassing the rhodamine labeled MSNs [DAPI signal (x-axis), density (y-axis)]. MSN without inulin or stool sample is represented as control (**a**) and shows no DAPI signal. An example of 4 samples in duplicates is displayed. DAPI fluorescence intensity percentages for each MSNs type are shown. Higher DAPI signal (bacteria signal) in the gate encompassing the rhodamine labeled MSNs was detected in the inulin-coupled hydrophilic MSNs (**c**) and inulin-coupled hydrophobic MSNs (**e**) with respect to the corresponding starting material (**b, d**).

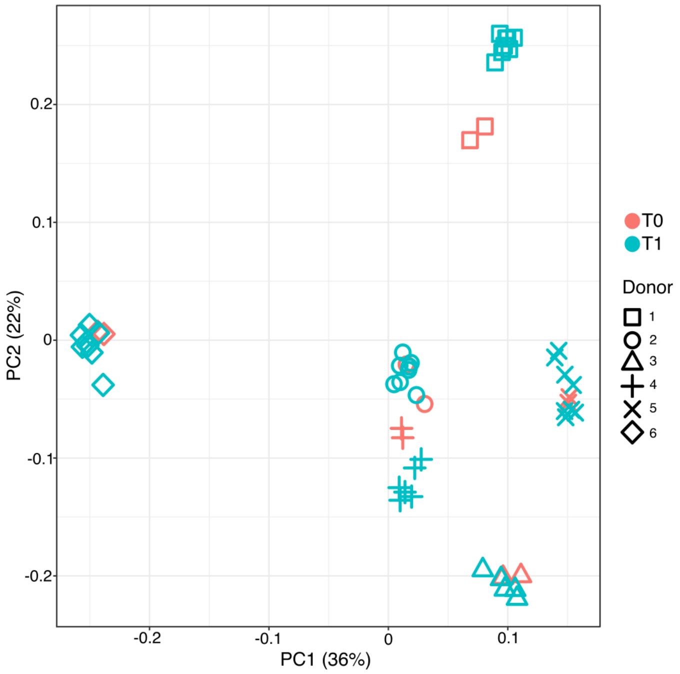

Supplementary Figure 3. Principal coordinates analysis ordination of 16S rRNA gene amplicon profiles from mesoporous silica nanoparticle (MSN) at 0 and 1 h in incubations.

Samples from total community at 0 (red) and 1 h (blue) are shown. Ordination shows significant grouping by donors, depicted with different symbols (PerMANOVA: p=0.001, n=60 samples).

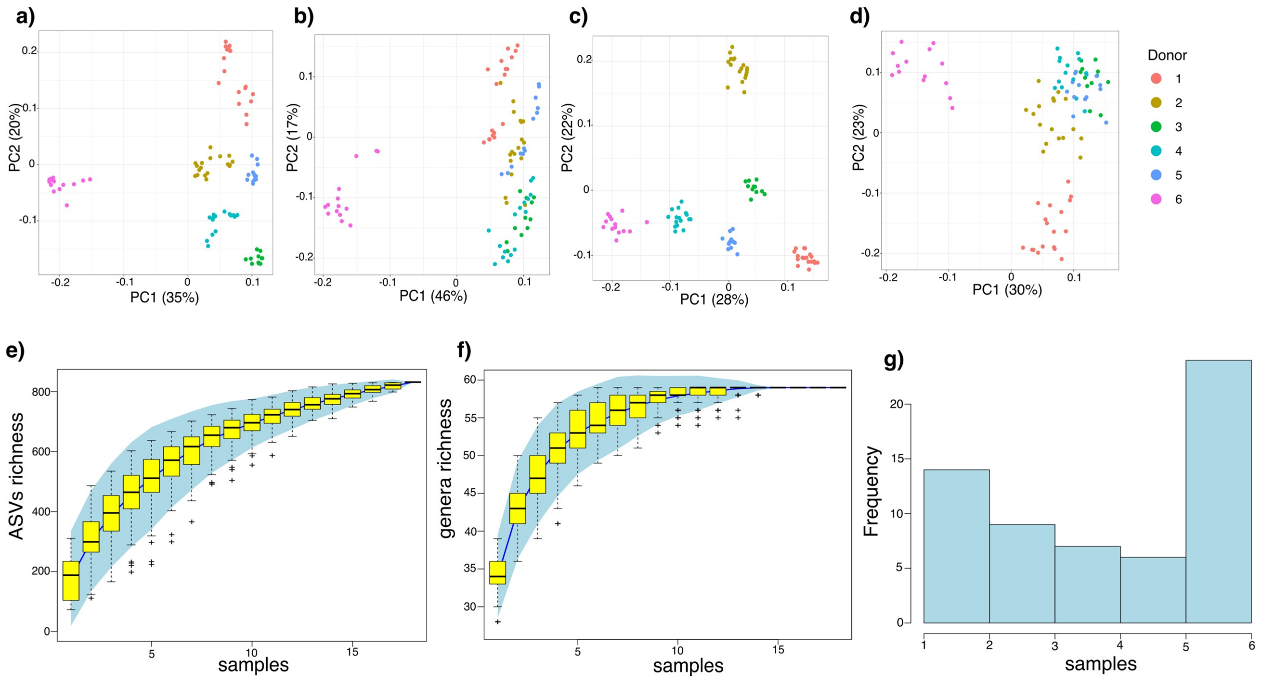

Supplementary Figure 4. Principal coordinates analysis ordination of 16S rRNA gene amplicon profiles from mesoporous silica nanoparticle (MSN) incubation experiments.

(a, b) Samples from total community, as well as from sorted inulin-grafted and ungrafted hydrophobic and hydrophilic MSNs, are shown in technical duplicate. Samples are colored by donor stool and show a clear clustering by donor. Principal coordinates analysis based on Bray-Curtis distances performed at (**a**) ASV and (**b)** genus levels (PerMANOVA: p=0.001; ANOSIM: p=0.001 for both ASV and genera comparisons, n=108 samples) and based on Bray-Curtis distances on binary (presence/absence) data performed at (**c**) ASV and (**d**) genus levels (PerMANOVA: p=0.001; ANOSIM: p=0.001 for both ASV and genus comparisons, n=108 samples) are shown. (**e**, **f)** Species accumulation curves at ASVs and genera level for the inulin-binding fraction of the microbiota of each MSNs type. (**g)** Histogram shows the prevalence of the most abundant genera (>1%) in the six samples.

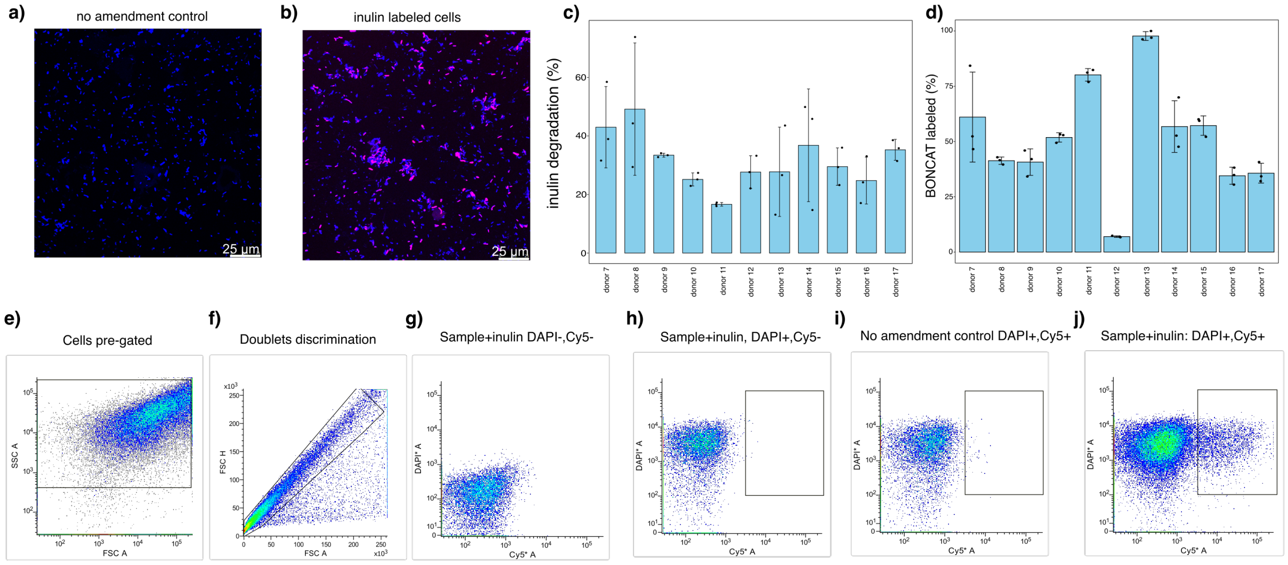

Supplementary Figure 5. **FACS-sorting gating strategy for BONCAT labelled cells.**

(**a**) No amendment control shows no BONCAT signal. (**b**) Active cells show a positive BONCAT signal (pink, Cy5) after 6 h incubation with inulin. All cells are stained with DAPI (blue). Both microscopy images were recorded using identical settings. **(c)** Results from the fructan assay shows inulin degradation by the total community (mean ± sd: 31.7 ± 13%, n=11 samples). Triplicates measurements are shown. (**d)** Percentage of positive BONCAT cells by donor (mean ± sd: 51.3 ± 24.3%, n=11 samples). Triplicates measurements are shown. Error bars represent standard deviation of the mean. (**e-h**) Bacteria were sorted with a FACS Melody cell sorter. The sorting gate was chosen to select the active fraction upon incubation with inulin. **e)** Bacteria were visualized using the forward scatter (FSC) and side scatter (SSC) and pre-gated, **f)** Doublets discrimination was performed to ensure that they were excluded from analysis to avoid false positive in the sorting experiments. Cy5 negative cells were used to select the gate: (**g**) unstained cells, (**h**) DAPI- stained cells, (**i**), no amendment control, (**j**), DAPI- and Cy5-labeled cells.

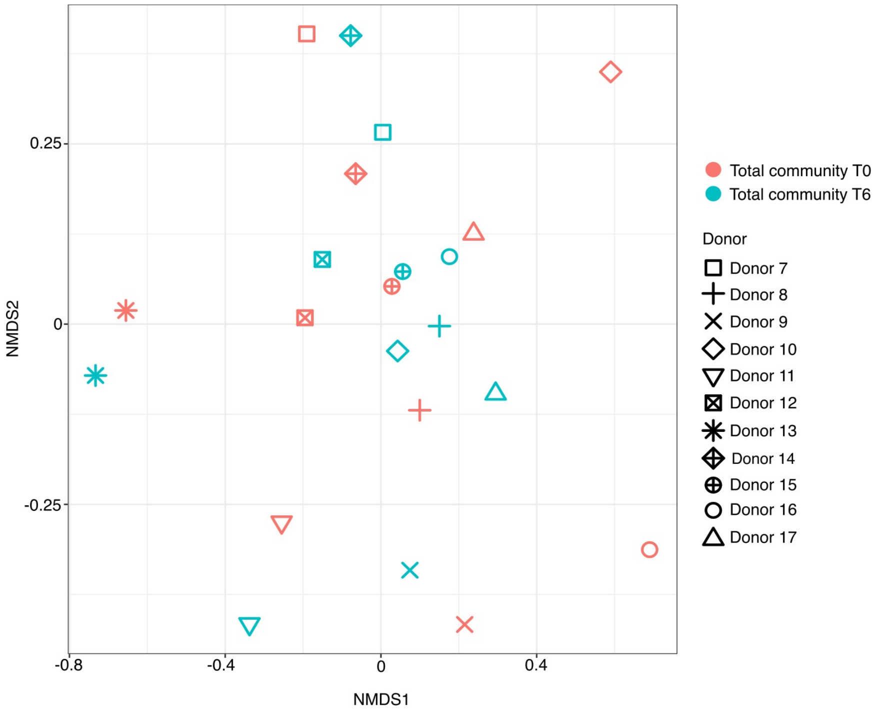

Supplementary Figure 6. Total community after inulin supplementation of 16S rRNA gene amplicon profiles.

No significant difference was detected in the total community comparing 0 h (red dots) and 6 h (blue dots) (PerMANOVA, p=0.591, n=22 samples). Ordination shows significant grouping by donors, depicted with different symbols (PerMANOVA: p=0.001, n=22 samples).

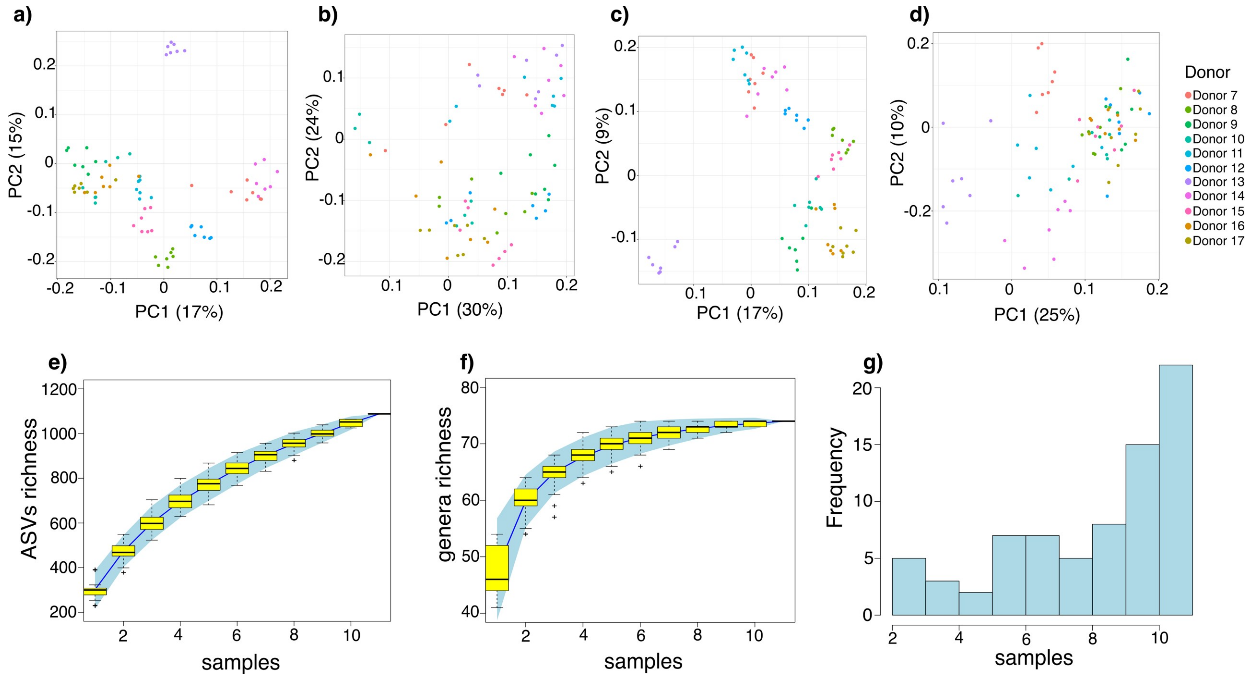

Supplementary Figure 7. Principal coordinates analysis ordination of 16S rRNA gene amplicon profiles from BONCAT incubation experiments after inulin supplementation.

(a,b) Samples from total community incubations, as well as from active fraction are shown. Samples are colored by donor and show a clear clustering by donor. Principal coordinates analysis based on Bray-Curtis distances performed at (**a**) ASV and (**b)** genus levels (PerMANOVA: p=0.001; ANOSIM: p=0.001 for both ASV and genus comparisons, n=77 samples) and based on Bray-Curtis distances on binary (presence/absence) data performed at (**c**) ASV and (**d)** genus levels (PerMANOVA: p=0.001; ANOSIM: p=0.001 for both ASV and genus comparisons, n=77 samples) are shown. (**e**, **f**) Species accumulation curves at ASV and genus level for the inulin-active fraction of the microbiota. **(g)** Histogram of the prevalence of the most abundant genera (>1%) in the eleven samples.

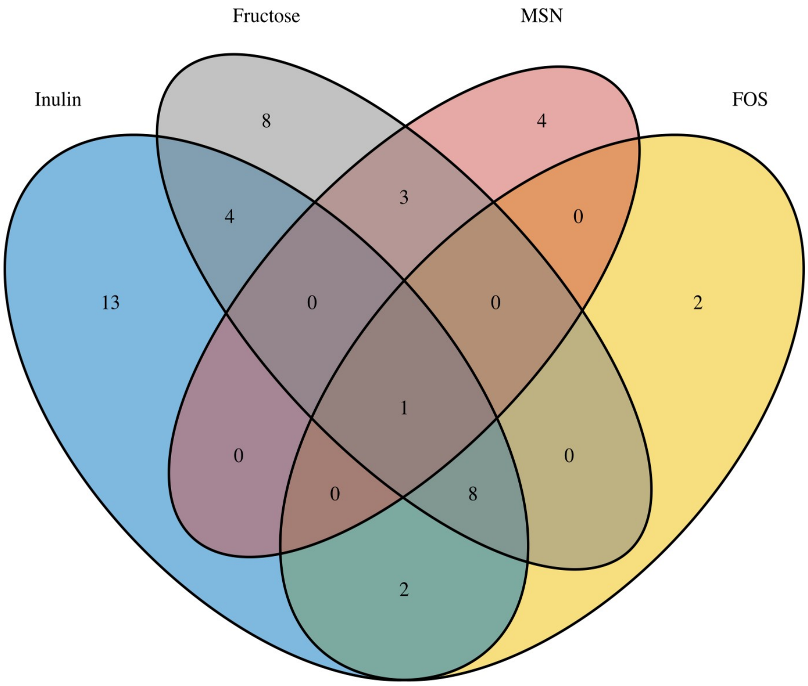

Supplementary Figure 8. **Venn diagram of enriched ASVs after incubation with different substrates and BONCAT-based sorting of translationally active microbial community members.**

Venn diagram shows shared and unique ASVs after inulin, FOS, fructose amendments that were enriched in the sorted fraction as well as the enrichments of MSN-FACS sorted cells. Venn diagram shows 13, 8, 4, and 2 unique ASVs for inulin, fructose, MSN, and FOS enrichments respectively. 11 ASVs were shared between inulin and FOS enrichments, 13 between inulin and fructose enrichments and 9 between FOS and fructose enrichments. 3 enriched ASVs from the binding experiments were shared with ASVs enriched after fructose amendment (ASV_2st_345: *Lachnospiraceae Blautia*, ASV_2ua_io6: *Bacteroidaceae Phocaeicola*, ASV_s62_9ct: *Lachnospiraceae Blautia*, ASV_sj9_cdm: *Ruminococcaceae Ruminococcus*) and 1 between binding experiments, inulin and FOS supplementation (ASV_2st_345= *Lachnospiraceae Blautia*). Details of unique and shared ASVs between the groups are shown in **Supplementary Table 5**.

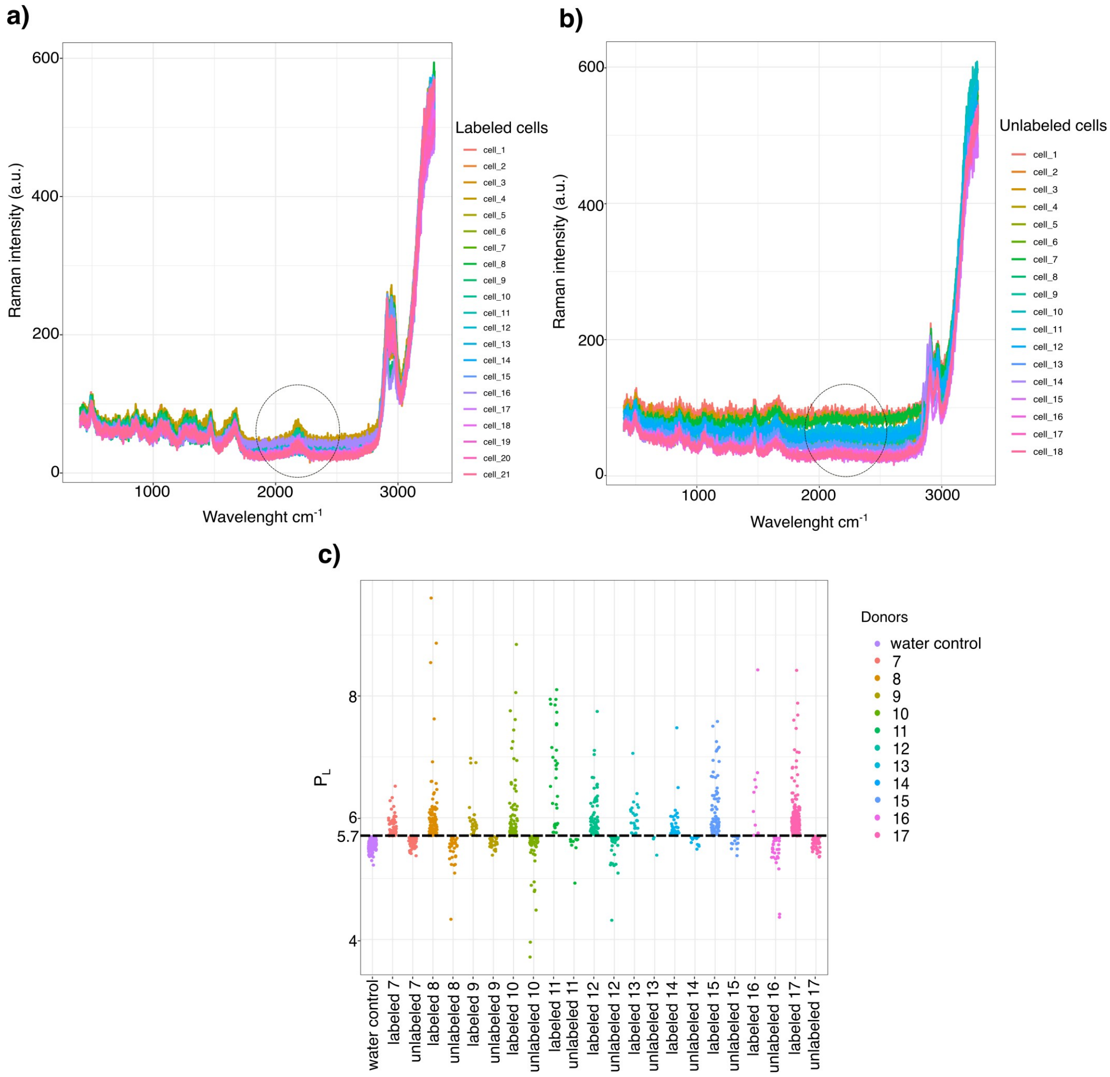

Supplementary Figure 9. Raman spectra of D-labeled and unlabeled cells in RACS.

(**a**) Example of Raman spectra of D-labeled and sorted cells. (**b)** Example of Raman spectra of unlabeled and rejected cells. The C-D peak region of the Raman spectrum (2,040-2,300 cm^{-1}) representing D incorporation is depicted by a circle. (**c**) P_L threshold was calculated as mean+2 sd of the water control (inulin in H_2O, n=116). The dashed line denotes the threshold P_L value adopted based on these controls (P_L = 5.7). Cells with a P_L > 5.7 were considered labeled and thus sorted, while cells with a $P_L \leq$ 5.7 were considered unlabeled and thus rejected. Dots depicted with different colors represent all cells analyzed for each donor. Numbers of labeled and unlabeled cells for each donor are shown in **Supplementary Table 6**.

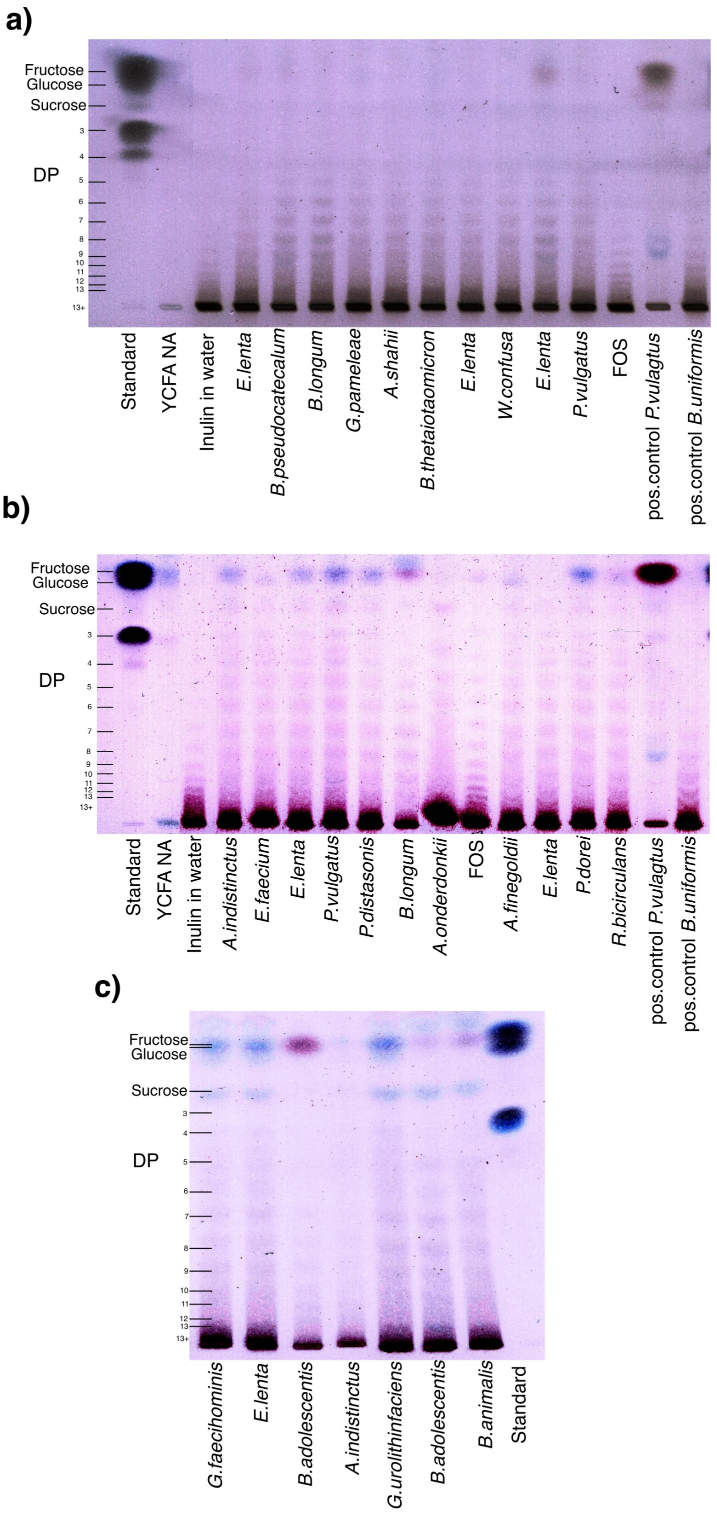

Supplementary Figure 10. Thin layer chromatography of RACS strains.

(**a**-**c**) Degree of polymerization (DP) distribution evaluation. Standard: fructose, glucose (DP 1), sucrose (DP 2), 1,1 kestotriose (DP 3) and 1,1,1 kestotetraose (DP 4). Inulin in water and YFCA were used as controls. Neither of these show the presence of monosaccharides or saccharides with a DP

less than 9. *Phocaeicola vulgatus* (DSM1447) and *Bacteroides uniformis* (DSM6597) were used as positive control strains. All strains tested show the capability of inulin degradation at different levels. Strains were sampled in their early stationary phase. **(a)** *Eggertella lenta* (donor 10), *Bifidobacterium pseudocatenulatum* (donor 10), *Bifidobacterium longum* (donor 10) *Gordonibacter pamelaeae* (donor 10), *Alistipes shahii* (donor 10), *Bacteroides thetaiotaomicron* (donor 11), *Eggerthella lenta* (donor 11), *Weissella confusa* (donor 12), *Eggertella lenta* (donor 12), *Phocaeicola vulgatus* (donor 12). (**b**) *Alistipes indistinctus* (donor 7), *Enterococcus faecium* (donor 7), *Eggertella lenta* (donor 7), *Phocaeicola vulgatus* (donor 8), *Parabacteroides distasonis* (donor 8), *Bifidobacterium longum* (donor 8), *Alistipes onderdonkii* (donor 8), *Alistipes finegoldii* (donor 9), *Eggertella lenta* (donor 9), *Phocaeicola dorei* (donor 9), *Ruminococcus bicirculans* (donor 9). (**c**) *Gordonibacter faecihominis* (donor 14), *Eggerthella lenta* (donor 14), *Bifidobacterium adolescentis* (donor 17), *Alistipes indistinctus* (donor 17), *Gordonibacter urolithinfaciens* (donor 17), *Bifidobacterium adolescentis* (donor 15), *Bifidobacterium animalis* (donor 15).

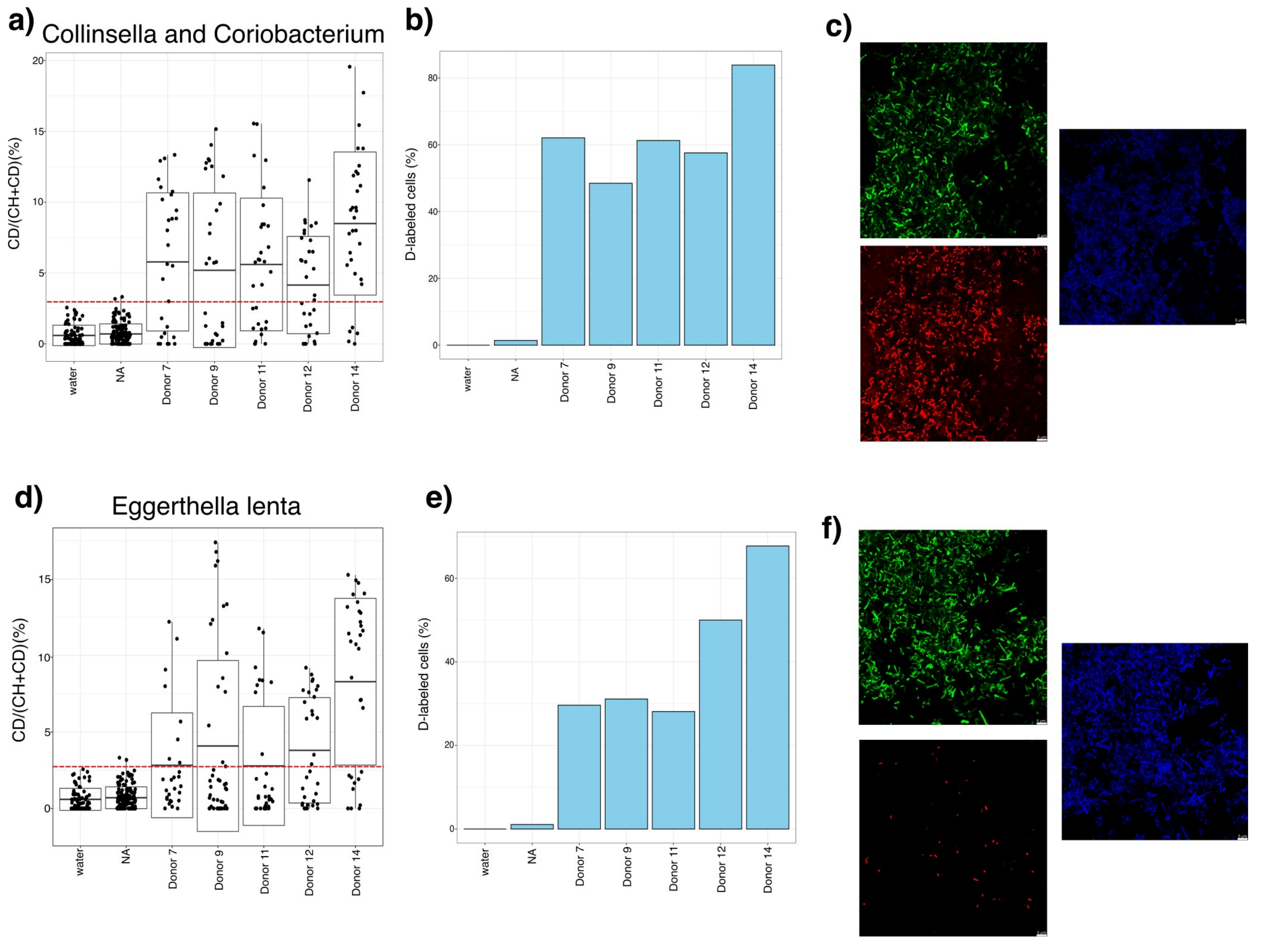

Supplementary Figure 11. Coriobacteriia show high metabolic activity after inulin supplementation: Raman-FISH experiments on stool samples amended with inulin revealed deuterium incorporation for both *Collinsella, Coriobacterium* and *Eggerthella lenta*.

(a) Raman measurements of FISH-identified *Collinsella, Coriobacterium* cells in stool samples from donors 7, 9, 11, 12, and 14 after 6 h incubation with inulin. The y-axis represents the percentage of active cells (%CD) based on deuterium incorporation. Each dot represents a random single cell measurement (water control: n=76 cells, nothing added NA n=142 cells, donor 7 n=29 cells, donor 9 n=33 cells, donor 11 n=31 cells, donor 12 n=33 cells, donor 14 n=31 cells). The red dashed line at 2.75% indicates the threshold for considering a cell labelled as previously explained. Boxplot: boxplot medians (center lines), interquartile ranges (box ranges), whisker ranges, scale bar 5 µm. (**b**) Percentage of deuterium-labeled *Collinsella, Coriobacterium* cells per donor. (**c**) Representative FISH image of a stool sample. The pictures show *Collinsella, Coriobacterium* stained with the

specific probes COR653 (red), all bacteria stained with EUB 338 I-III (green), and DAPI staining (blue). (**d)** Raman measurements of positive *Eggerthella lenta* cells in stool samples from donors 7, 9, 11, 12, and 14 after 6 h incubation with inulin (water control: n=76 cells, nothing added NA n=142 cells, donor 7 n=27 cells, donor 9 n=45 cells, donor 11 n=32 cells, donor 12 n=32 cells, donor 14 n=31 cells). **(e**) Percentage of deuterium-labeled *Eggerthella lenta* cells per donor. **(f)** Representative FISH image of a stool sample. The pictures show *Eggerthella lenta* stained with the specific probes E.lenta 194 (red), all bacteria stained with EUB 338 I-III (green), and DAPI staining (blue).

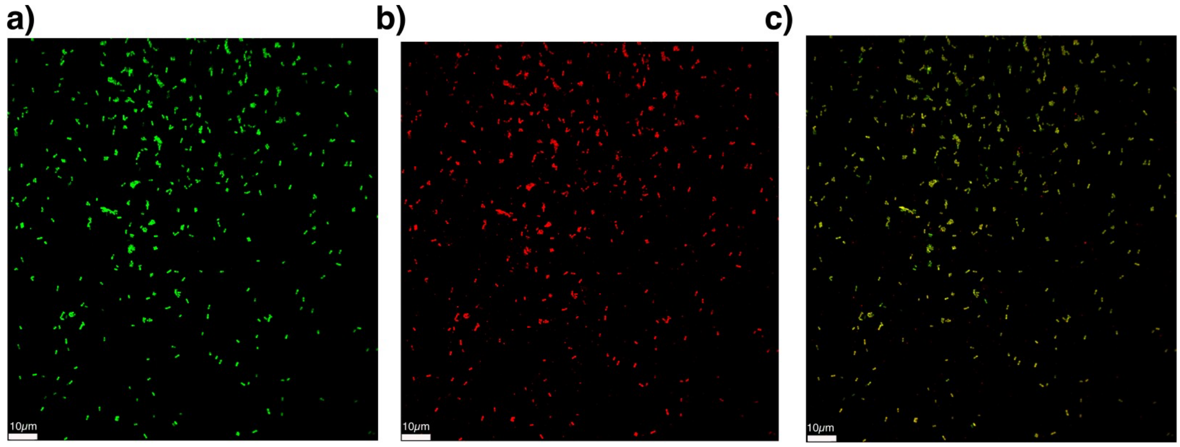

Supplementary Figure 12. FISH of *Eggerthella lenta* isolated with RACS.

(**a**) *Eggerthella lenta* isolated with RACS stained with probes for all bacteria (EUB 338 I-III; green) and (**b**) the specific probe E.len194 (red). (**c**) overlay of signals.

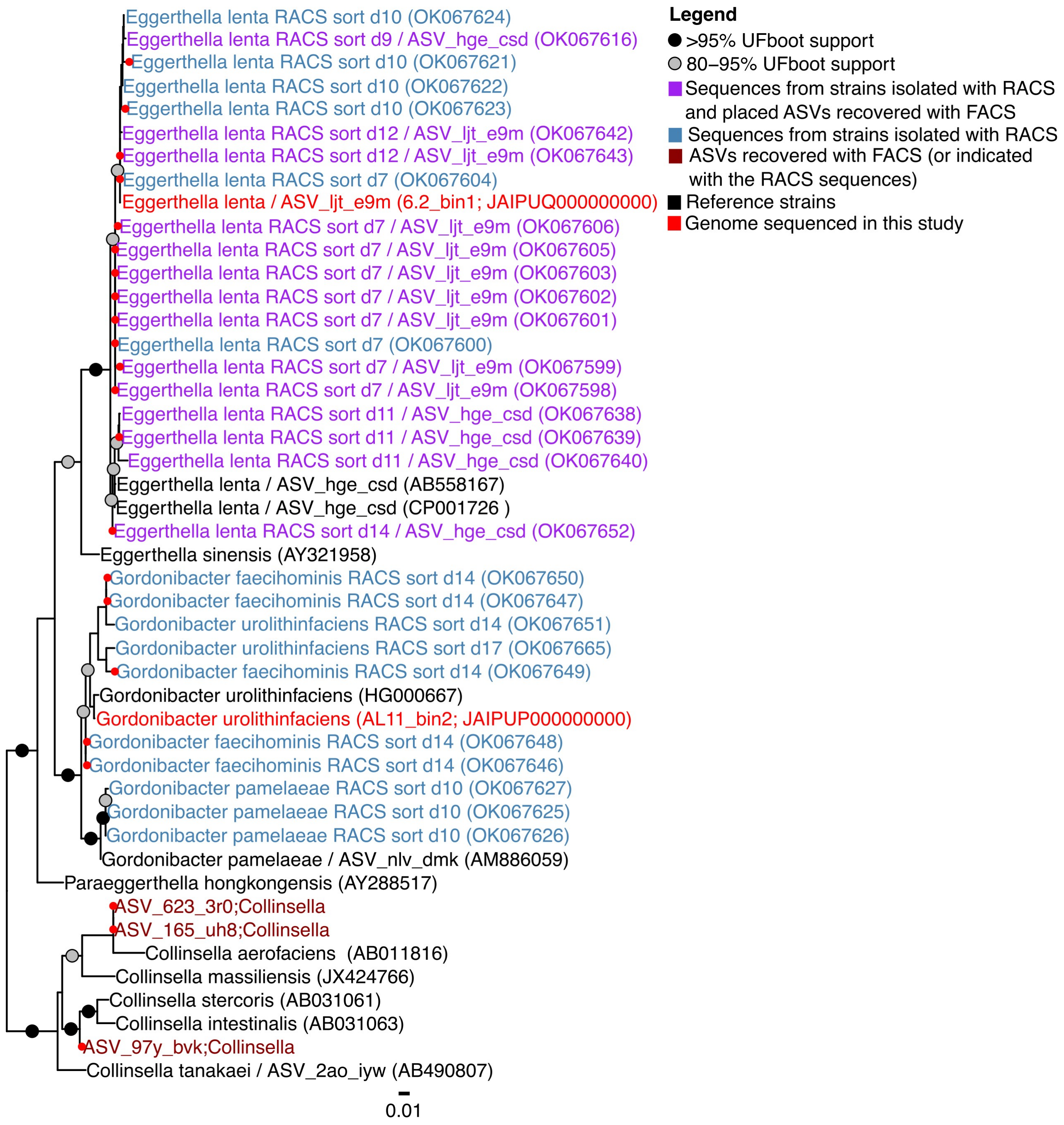

Supplementary Figure 13. Phylogenetic tree of the class *Coriobacteriia* members build with 16S rRNA gene amplicon ASV sequences from FACS-sorted cells and sequences from the strains isolated with RACS. Black dots indicate support values > 95% and grey dot between 80 and 95%, red dots indicate the sequences shorter than 1200 nucleotides placed in the tree. Sequences from the strains isolated with RACS are indicate in blue, ASVs recovered with FACS from both BONCAT and MSN sort experiments are indicated in purple or indicated with the RACS sequences, black labels indicate the type strains used as reference sequences and in red are indicated the 16S rRNA sequences

recovered from genomes of *Gordonibacter urolithinfaciens* AL-11 and *Eggerthella lenta* 6.2 sequenced in this study. Accession numbers are indicated in parenthesis.

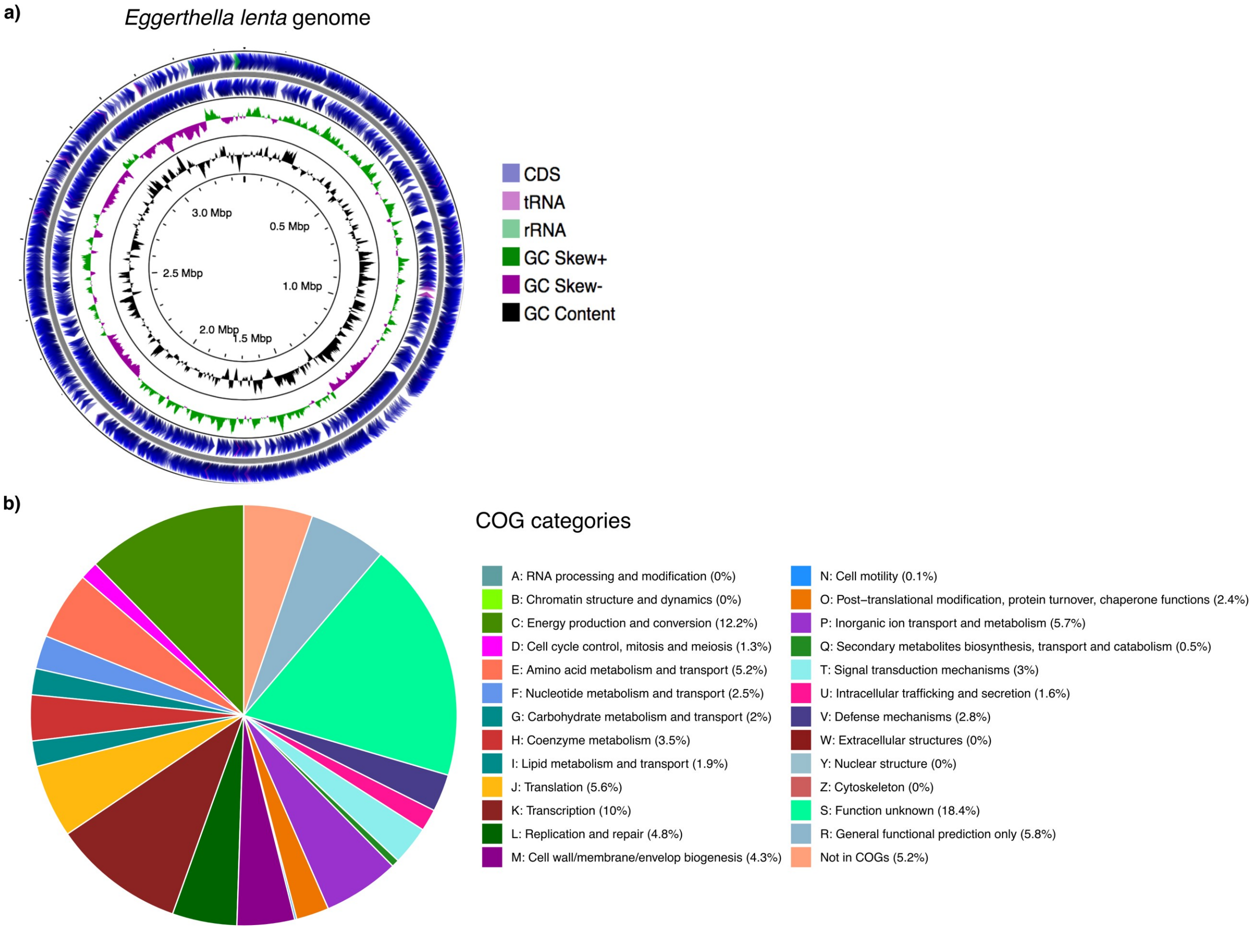

Supplementary Figure 14. Genome representation of *Eggerthella lenta* 6.2.

(a) Circular map of the *Eggerthella lenta* genome. Circles from the outside to the inside show the positions of protein-coding genes (blue), tRNA genes (violet) and rRNA genes (light green) on the positive (circle 1), and negative (circle 2) strands. Circles 3 and 4 show plots of GC content and GC skew plotted as the deviation from the average for the entire sequence. The genome was visualized with GCView Server. **(b)** Percentage of genes associated with the respective COG functional category. Genome annotations revealed a predicted 2.0% of genes involved in carbohydrate metabolism and transport for *E. lenta* 6.2, including several different glycoside hydrolases, ABC transporters, permeases and sugar phosphotransferase systems (PTS).

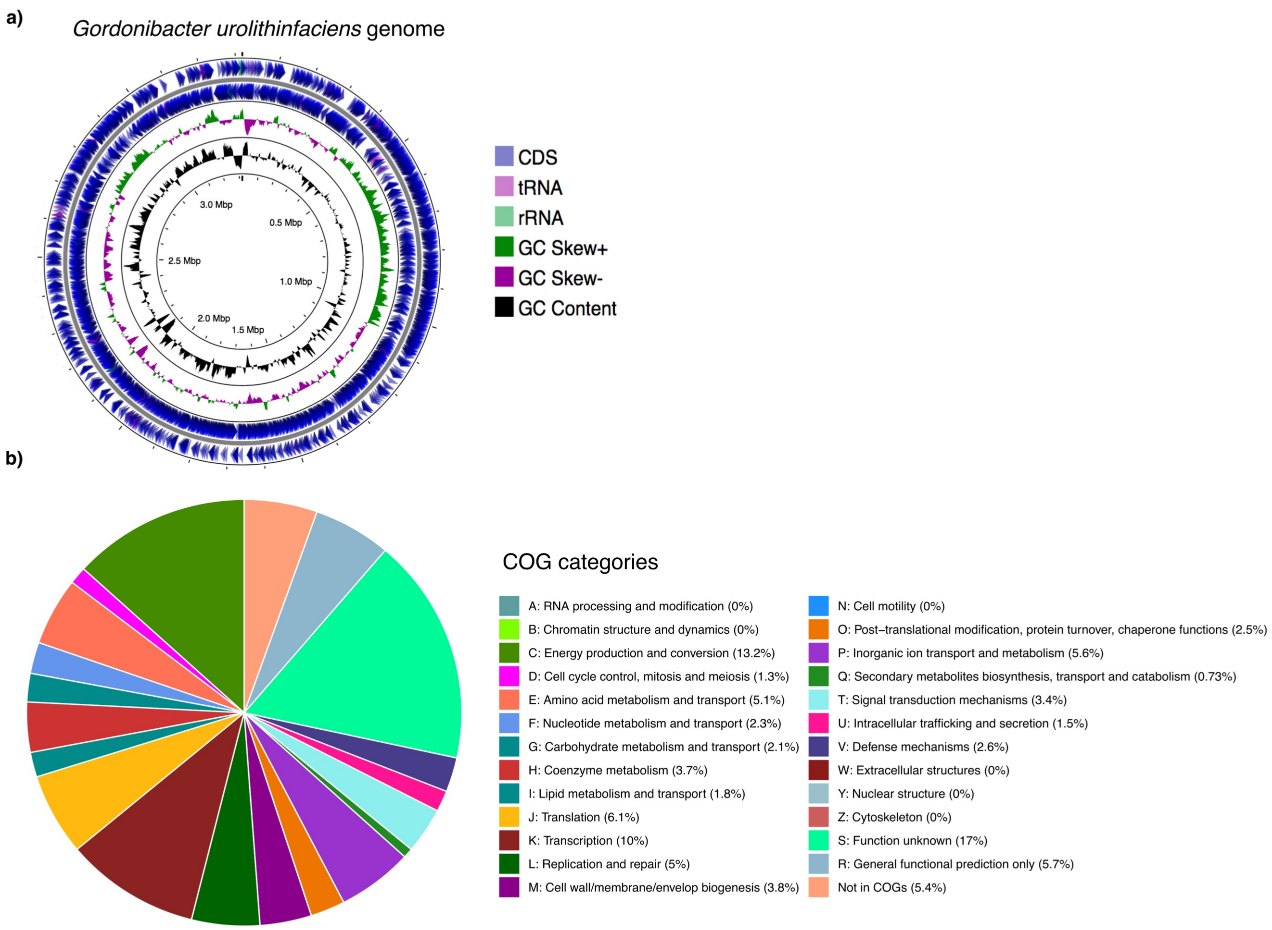

Supplementary Figure 15. Genome representation of *Gordonibacter urolithinfaciens* AL-11.

(a) Circular map of the *Gordonibacter urolithinfaciens* genome. Circles from the outside to the inside show the positions of protein-coding genes (blue), tRNA genes (violet) and rRNA genes (light green) on the positive (circle 1), and negative (circle 2) strands. Circles 3 and 4 show plots of GC content and GC skew plotted as the deviation from the average for the entire sequence. The genome was visualized with GCView Server. (**b)** Percentage of genes associated with the respective COG functional categories. Genome annotations revealed a predicted and 2.1% of genes involved in carbohydrate metabolism and transport for *G. urolithinfaciens* AL-11, including several different glycoside hydrolases, ABC transporters, permeases and sugar phosphotransferase systems (PTS).

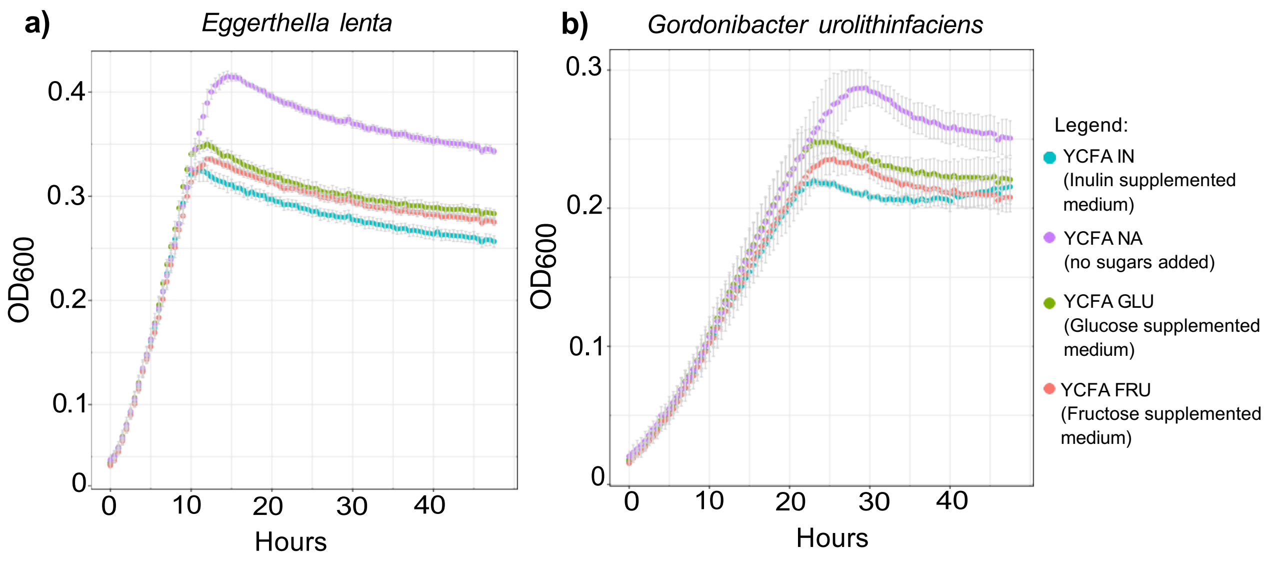

Supplementary Figure 16. Growth curves of *Eggerthella lenta* and *Gordonibacter urolithinfaciens* isolated with RACS after inulin, glucose, or fructose supplementation.

(a) *Eggerthella lenta* and **(b)** *Gordonibacter urolithinfaciens* were grown in inulin (light blue), glucose (green) and fructose (red) supplemented YCFA-medium and in YCFA-medium without any other sugar added (NA, violet) that was used as control. The medium was prepared using the recipe from DSMZ 1611 modified with the addition of 10 mg/ml arginine. The growth curves are expressed as mean values (three replicates) ± sd of optical densities measured at 600 nm.

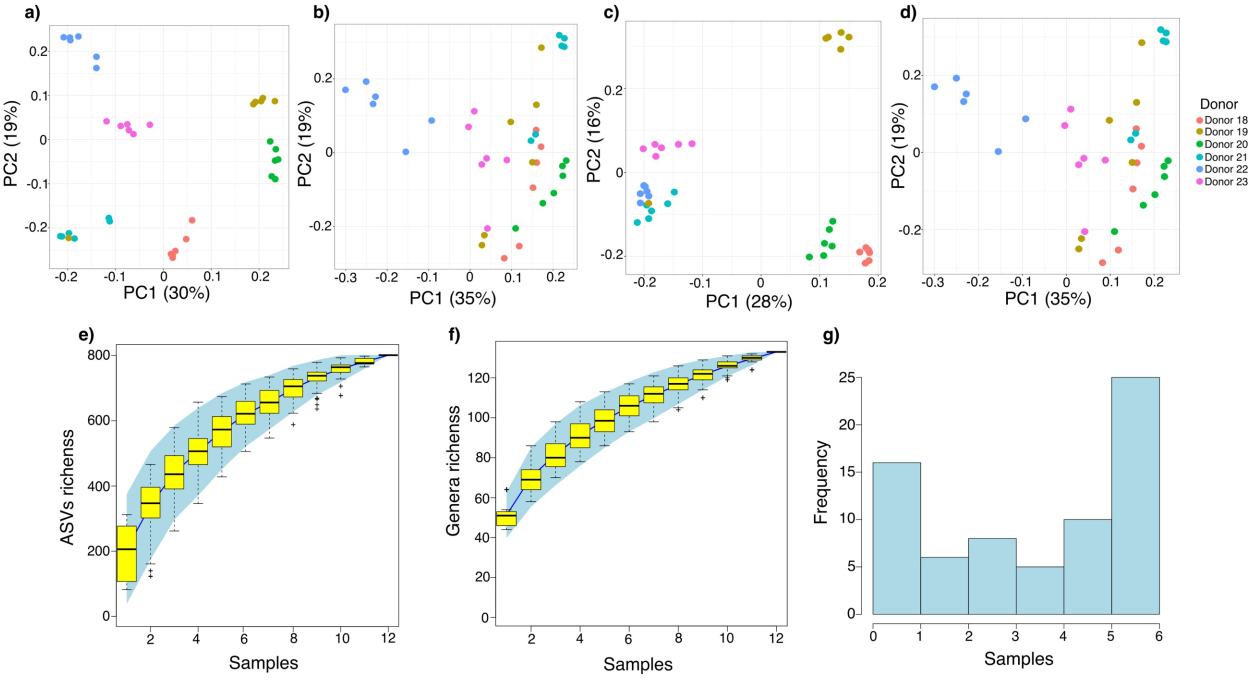

Supplementary Figure 17. Principal coordinates analysis ordination of 16S rRNA gene amplicon profiles from BONCAT incubation experiments after XOS supplementation.

(a,b) Samples from total community incubations, as well as from active fraction are shown. Samples are colored by donor and show a clear clustering by donor. Principal coordinates analysis based on Bray-Curtis distances performed at (**a**) ASV and (**b**) genus levels (PerMANOVA: p=0.001; ANOSIM: p=0.001 for both ASV and genus comparisons, n=36 sample) and based on Bray-Curtis distances on binary (presence/absence) data performed at (**c**) ASV and (**d**) genus levels (PerMANOVA: p=0.001; ANOSIM: p=0.001 for both ASV and genus comparisons, n=36 sample) are shown. (**e**, **f**) Species accumulation curves at ASV and genus level for the XOS-active fraction of the microbiota. (g) Histogram of the prevalence of the most abundant genera (>1%) in the six samples.

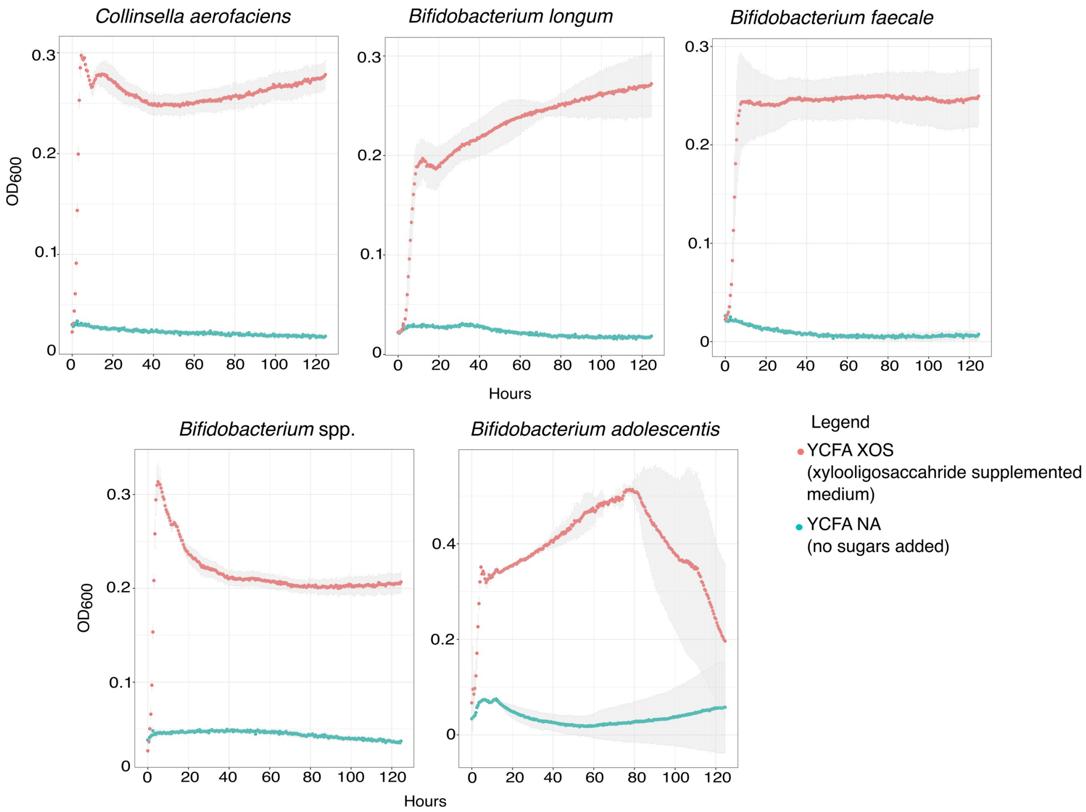

Supplementary Figure 18. Growth curves of representative strains isolated with RACS after XOS supplementation.

Growth curves of the most representative strains isolated with RACS. Representative strains were selected choosing all the different species isolated. All strains were grown in XOS supplemented YCFA-medium (red) and in YCFA-medium without XOS and any other sugar added (NA, blue) that was used as control. The medium was prepared using the recipe from DSMZ 1611. The growth curves are expressed as mean values (three replicates) ± sd of optical densities measured at 600 nm.

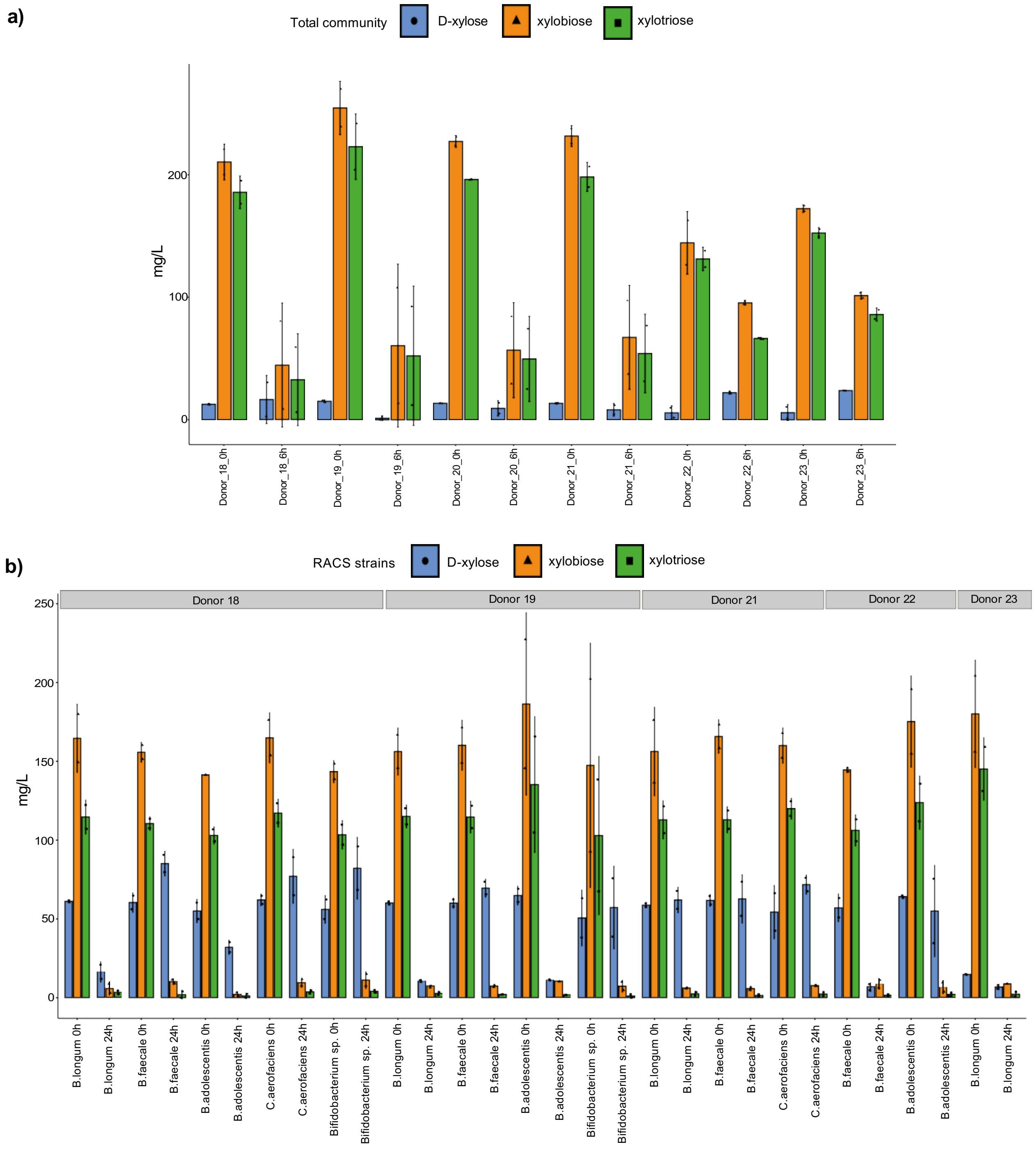

Supplementary Figure 19. HPAEC measurements of D-xylose, xylobiose and xylotriose for the total community and each RACS isolates for each donor.

(**a**) HPAEC measurement of the stool sample at 0 and 6 h (total community). A statistical significance decrease was observed after 6 h for xylobiose (0 h: 206.7 ± 40.7, 6 h: 70.9 ± 37.4, n=12 samples, p<0.0001, Student t-test) and xylotriose (0 h: 181.1 ± 33.5, 6 h: 56.8 ± 30.3, p<0.0001, n=12 samples, Student t-test) but not for D-xylose (0 h: 11.1 ± 4.7, 6 h: 13.6 ± 10.5, p=0.475, n=12 samples, Student

t-test). (**b**) RACS-sorted strains were grown at 24 h in XOS-supplemented media and compare to 0 h time point. Student t-test or Wilcox test comparing 0h with 24h; D-xylose (0 h: 56.14 ± 13.11, 24 h: 47.15 ±30.74, p=0.876, n=60 samples), xylobiose (0 h: 160.21 ± 25.32, 24 h: 7.69 ± 2.95, p<0.0001, n=60 samples), xylotriose (0 h: 115.88 ± 18.35, 24 h: 2.32± 1.41, p<0.0001, n=60 samples). Student t-test comparing each bacteria isolated: *Bifidobacterium longum*, D-xylose (0 h: 48.78 ± 7.42, 24 h: 24.02 ± 11.3, p= 0.724, n=8 samples), xylobiose (0 h: 164.4 ± 7.78, 24 h: 7 ± 0.71, p<0.0001, n=8 samples), xylotriose, (0 h: 122 ± 6.18, 24 h: 2.76 ± 0.47, p<0.0001, n=8 samples); *Bifobacterium faecale*, D-xylose (0 h: 59.87 ± 1.69, 24 h: 56.07 ± 11.59, p<0.0001, n=8 samples), xylobiose (0 h: 156.6 ± 3.98, 24 h: 8 ± 0.83, p= 0.0005, n=8 samples), xylotriose (0 h: 111.1 ± 2.54, 24 h: 71.77± 0.43, p= 0.0001, n=8 samples), *Bifobacterium adolescentis*, D-xylose (0 h: 61.41 ± 2.64, 24 h: 32.58 ± 9.96, p= 0.013, n=8 samples), xylobiose (0 h: 167.7 ± 14.58, 24 h: 6.36 ± 1.7, p= 0.0002, n=8 samples), xylotriose (0 h: 120.7 ± 10.39, 24 h: 1.72 ± 0.46, p<0.0001, n=8 samples), *Bifobacterium* sp., D-xylose (0 h: 53.75 ± 11.87, 24 h: 69.36 ± 13.26, p= 0.281, n=8 samples), xylobiose (0 h: 145.5 ± 22.49, 24 h: 9.32 ± 2.17, p= 0.003, n=8 samples), xylotriose (0 h: 103.2 ± 14.72, 24 h: 2.61 ± 1.03, p= 0.001, n=8 samples), *Collinsella aerofaciens*, D-xylose (0 h: 58.26 ± 5.44, 24 h: 74.48 ± 7.7, p= 0.999, n=8 samples), xylobiose (0 h: 162.5 ± 5.77, 24 h: 8.68 ± 1, p= 0.016, n=8 samples), xylotriose (0 h: 118.6 ± 3.26, 24 h: 3.11 ± 0.73, p= 0.035, n=8 samples). Error bars represent standard deviation of the mean. Each dot represents a single measurement (samples were measured in duplicates, n=60 for RACS strains and n=24 for total community). D-xylose (blue), xylobiose (orange) and xylotriose (green).

Supplementary Tables

Supplementary Table 1. List of unique and shared ASVs of enrichments detected after inulin, FOS, fructose, and MSN amendments.

	Unique ASVs	Taxa
**Inulin**	ASV_27v_mrq	Unclassified *Lachnospiraceae*
	ASV_29g_ajq	*Lachnospiraceae Anaerostipes*
	ASV_29p_drg	Unclassified *Lachnospiraceae*
	ASV_5z7_fi0	*Lachnospiraceae Anaerostipes*
	ASV_64u_lsr	*Lachnospiraceae Roseburia*
	ASV_6fr_khd	*Sutterellaceae Parasutterella*
	ASV_9eq_kr6	*Streptococcaceae Streptococcus*
	ASV_fcp_dbf	*Corynebacteriaceae Corynebacterium*
	ASV_fqm_8pm	*Streptococcaceae Streptococcus*
	ASV_jvz_svl	Unclassified *Lachnospiraceae*
	ASV_nw9_c4l	*Lachnospiraceae Anaerostipes*
	ASV_p02_b8f	*Lachnospiraceae Blautia*
	ASV_pxk_1ex	*Lachnospiraceae Anaerostipes*
**MSN**	ASV_t5i_a3k	*Veillonellaceae Dialister*
	ASV_ter_b2k	*Eggerthellaceae Raoultibacter*
	ASV_tg8_il9	*Lachnospiraceae Roseburia*
	ASV_kp7_w4p	*Selenomonadaceae Mitsukella*
**Fructose**	ASV_76c_zrb	*Clostridiales*
	ASV_avd_9fb	*Desulfovibrionaceae Desulfovibrio*
	ASV_cns_p1y	*Ruminococcaceae Gemmiger*
	ASV_dqn_jls	*Bifidobacteriaceae Bifidobacterium*
	ASV_ic9_xvj	*Bifidobacteriaceae Bifidobacterium*
	ASV_ram_s25	*Bacteroidaceae Phocaeicola*
	ASV_sy7_30v	*Lachnospiraceae Kineothrix*
	ASV_t9m_xq7	*Bacteroidaceae Phocaeicola*
**FOS**	ASV_6ea_nz7	*Lachnospiraceae Blautia*
	ASV_a7l_qve	Unclassified *Lachnospiraceae*
	**Shared ASVs**	**Taxa**
**Inulin-FOS**	ASV_165_uh8	*Coriobacteriaceae Collinsella*
	ASV_2st_345	*Lachnospiraceae Blautia*
	ASV_4ma_8bd	*Lachnospiraceae Agathobacter*
	ASV_82p_r33	*Bifidobacteriaceae Bifidobacterium*
	ASV_ap2_sf2	*Erysipelotrichaceae Clostridium_XVIII*
	ASV_dx5_pax	*Erysipelotrichaceae Faecalibacillus*
	ASV_e9p_tub	*Lachnospiraceae Blautia*
	ASV_hcf_4f5	*Lachnospiraceae Blautia*
	ASV_hwd_896	*Lachnospiraceae Anaerobutyricum*
	ASV_jbt_n3b	Unclassified *Lachnospiraceae*
	ASV_ski_ka3	*Lachnospiraceae Blautia*
**Inulin-Fructose**	ASV_165_uh8	*Coriobacteriaceae Collinsella*
	ASV_2st_345	*Lachnospiraceae Blautia*
	ASV_623_3r0	*Coriobacteriaceae Collinsella*
	ASV_7s4_sc0	*Sutterellaceae Parasutterella*
	ASV_82p_r33	*Bifidobacteriaceae Bifidobacterium*
	ASV_dx5_pax	*Erysipelotrichaceae Faecalibacillus*
	ASV_e21_cmv	*Erysipelotrichaceae Amedibactrium*
	ASV_e9p_tub	*Lachnospiraceae Blautia*
	ASV_hcf_4f5	*Lachnospiraceae Blautia*
	ASV_hwd_896	*Lachnospiraceae Anaerobutyricum*
	ASV_jbt_n3b	Unclassified *Lachnospiraceae*
	ASV_kaw_sk8	*Ruminococcaceae Ruminococcus*
	ASV_ski_ka3	*Lachnospiraceae Blautia*
**Inulin-MSN**	ASV_2st_345	*Lachnospiraceae Blautia*
**FOS-Fructose**	ASV_165_uh8	*Coriobacteriaceae Collinsella*
	ASV_2st_345	*Lachnospiraceae Blautia*
	ASV_82p_r33	*Bifidobacteriaceae Bifidobacterium*
	ASV_dx5_pax	*Erysipelotrichaceae Faecalibacillus*
	ASV_e9p_tub	*Lachnospiraceae Blautia*
	ASV_hcf_4f5	*Lachnospiraceae Blautia*
	ASV_hwd_896	*Lachnospiraceae Anaerobutyricum*
	ASV_jbt_n3b	Unclassified *Lachnospiraceae*
	ASV_ski_ka3	*Lachnospiraceae Blautia*
**FOS-MSN**	ASV_2st_345	*Lachnospiraceae Blautia*
**Fructose-MSN**	ASV_2st_345	*Lachnospiraceae Blautia*
	ASV_2ua_io6	*Bacteroidaceae Phocaeicola*

ASV_s62_9ct	*Lachnospiraceae Blautia*
ASV_sj9_cdm	*Ruminococcaceae Ruminococcus*

Supplementary Table 2. D-labeled and unlabeled cells measured during RACS for each donor after inulin supplementation. Total number of cells sorted and colonies recovered are shown. Recovery represents the percentage of colonies recovered from the labeled cells.

	**Donors**										
	**7**	**8**	**9**	**10**	**11**	**12**	**13**	**14**	**15**	**16**	**17**
**D-labeled**	76	91	28	81	30	77	24	33	74	11	182
**Unlabeled**	56	37	31	53	9	29	3	13	14	27	45
**Total**	132	128	59	134	39	106	27	46	88	38	227
**Labeled (%)**	57%	71%	47%	60%	76%	72%	88%	71%	84%	29%	80%
**Colonies recovered (n)**	11	6	4	19	4	4	0	7	12	0	5
**Recovery (%)**	14.4%	6.6%	14.3%	23.4%	13.3%	5.2%	0%	21.2%	16.2%	0%	2.7%

Supplementary Table 3. Characteristics of isolates for each donor after inulin supplementation. Percentage of inulin used (detected concentration/starting concentration × 100) as determined by the fructan assay, and the degree of polymerization evaluation performed with thin layer chromatography (TLC) is shown. 13+ indicates that the TLC resolving limit is degree of polymerization (DP) 13. Monosaccharide concentration was measured with the fructan assay, and the type of monosaccharide released was determined by TLC. Growth capability on rich media YCFA supplemented with glucose, inulin or no addition of sugars is shown. Maximum OD_{600} detected in liquid culture during growth in inulin media is shown.

**Strains**	**Donor**	**Inulin used (%)**	**DP range**	**Monosaccharide detected by fructan assay mg/ml**	**Monosaccharides detected by TLC**	**Growth on YCFA+G£**	**Growth on YCFA+IN$**	**Growth on YCFA NA***	**Max OD_{600} YCFA IN**	**Max OD_{600} YCFA NA**
Alistipes indistinctus	7	26.05	4-13+	0.2	glucose	+	+	+/-	0.2	0.05
Enterococcus faecium	7	43.5	4-13+	0.2	glucose	+	+	+/-	0.15	0.1
Eggerthella lenta	7	29.3	4-13+	0.2	glucose	+	+	+	0.1	0.04
Phocaeicola vulgatus	8	38.4	3-13+	0.3	glucose	+	+	-	0.3	0.08
Parabacteroides distasonis	8	30.5	4-13+	0.2	glucose	+	+	-	0.6	0.17
Bifidobacterium longum	8	84	5-13+	0.3	glucose, fructose	+	+	+	0.17	0.13
Alistipes onderdonkii	8	5.3	6-13+	0.5	-	+	+	+	0.18	0.24
Alistipes finegoldii	9	28.8	5-13+	0.4	glucose	+	+	+/-	0.1	0.08
Eggerthella lenta	9	39.8	5-13+	0.5	-	+	+	+/-	0.09	0.05
Phocaeicola dorei	9	24.7	4-13+	0.14	glucose	+	+	+	0.1	0.02
Ruminococcus bicirculans	9	45	4-13+	0.05	fructose	+	+	+/-	0.15	0.005
Bifidobacterium pseudocatenulatum	10	59.6	4-13+	0	fructose	+	+	-	0.06	0.03
Eggerthella lenta	10	41.3	5-13+	0	fructose	+	+	+/-	0.09	0.05
Bifidobacterium longum	10	65.9	4-13+	0.07	-	+	+	+/-	0.08	0.05
Gordonibacter pameleae	10	38.1	4-13+	0.16	glucose	+	+	+/-	0.05	0.04
Alistipes shahii	10	32.4	4-13+	0.01	-	+	+	+/-	0.13	0.09
Bacteroides thetaiotaomicron	11	30.4	5-13+	0	-	+	+	-	0.06	0.005
Eggerthella lenta	11	24.8	5-13+	0	-	+	+	+/-	0.05	0.03
Weisella confusa	12	0.1	5-13+	0.08	-	+	+	-	0.09	0.03
Eggerthella lenta	12	51.8	4-13+	0.08	fructose	+	+	-	0.07	0.05
Phocaeicola vulgatus	12	9.4	5-13+	0.47	-	+	+	+/-	0.05	0.01
Gordonibacter faecihominis	14	33.5	4-13+	0.007	glucose	+	+	+/-	0.08	0.03
Eggerthella lenta	14	33	4-13+	0	glucose	+	+	+/-	0.07	0.03
Bifidobacterium adolescentis	15	59.3	4-13+	0.93	fructose	+	+	+	0.4	0.15
Bifidobacterium animalis	15	7.8	4-13+	0	fructose	+	+	+	0.02	0.02
Bifidobacterium adolescentis	17	48.5	6-13+	0	fructose	+	+	+/-	0.2	0.1
Alistipes indistinctus	17	32	6-13+	0	-	+	+	+/-	0.07	0.005
Gordonibacter urolithinfaeciens	17	37.8	4-13+	0	glucose	+	+	+/-	0.07	0.02

Legend: £ YCFA-glucose, $YCFA-inulin, *YCFA-no amendment, + positive growth, - negative growth

Supplementary Table 4. ANI genome comparisons.

Average Nucleotide Identity (ANI) comparisons were calculated between *E. lenta* 6.2, *G. urolithinfaciens* AL-11 and reference genomes deposited in NCBI.Average nucleotide identity (ANI) comparison of isolates 6.2 and AL-11 confirmed their assignment to strains of *E. lenta* (98.34% ANI to the type strain *E.lenta* 1899 B/DSM 2243) within the genus *Eggerthella*, and *G. urolithinfaciens* (98.69% ANI to the type strain *G. urolithinfaciens* DSM 27213) within the genus *Gordonibacter*, respectively.

**Comparison for *E.lenta* 6.2**	**OrthoANI u-value (%)**
Eggerthella lenta GCA_000024265.1	98.34
Eggerthella sinensis CGA_003339815.1	84.24
Eggerthella guodeyninii GCA_009834925.2	88.26
Eggerthella timoniensis GCA_900184265.1	87.07
**Comparison for *Gordonibacter* AL-11**	
Gordonibacter pameleae GCA_000210055.1	92.95
Candidatus Gordonibacter avicola GCA_019113915.1	79.75
Gordonibacter urolithinfaciens GCA_902386185.1	98.69
Gordonibacter massiliensis GCA_902386835.1	85.23

Supplementary Table 5. D-labeled and unlabeled cells measured during RACS for each donor after XOS supplementation. Total number of cells sorted and colonies recovered are shown. Recovery represents the percentage of colonies recovered from the labeled cells.

	Donors					
	**18**	**19**	**20**	**21**	**22**	**23**
**D-labeled**	26	8	4	8	14	1
**Unlabeled**	360	29	154	91	102	118
**Total**	386	37	158	99	116	119
**Labeled (%)**	6.7%	21.6%	2.5%	8%	12%	0.84%
**Colonies recovered (n)**	25	8	1	8	11	1
**Recovery (%)**	96.1%	100%	25%	100%	78.5%	100%

Supplementary Table 6. Isolate characteristics for each donor after XOS supplementation.

Maximum OD_{600} detected in liquid culture during growth in XOS compared to the medium without addition of sugars is shown.

Strains	Donor	Max OD_{600} YCFA XOS	Max OD_{600} YCFA NA
Bifidobacterium longum	18	0.45	0.02
Bifobacterium faecale	18	0.41	0.04
Bifidobacterium adolescentis	18	0.51	0.07
Collinsella aerofaciens	18	0.32	0.06
Bifidobacterium spp.	18	0.31	0.05
Bifidobacterium longum	19	0.27	0.03
Bifobacterium faecale	19	0.29	0.15
Bifidobacterium adolescentis	19	0.33	0.04
Bifidobacterium longum	21	0.30	0.23
Bifobacterium faecale	21	0.29	0.04
Collinsella aerofaciens	21	0.29	0.03
Bifobacterium faecale	22	0.25	0.02
Bifidobacterium adolescentis	22	0.38	0.28
Bifidobacterium longum	23	0.40	0.10
