# Supplementary material for: Identification of inulin-responsive bacteria in the gut microbiota via multi-modal activity-based sorting
Source: Nat Commun. 2023 Dec 14;14:8210. doi: 10.1038/s41467-023-43448-z (PMC10721620; doi:10.1038/s41467-023-43448-z)
Supplement: Supplementary file 3 — Description of Additional Supplementary Files [file 41467_2023_43448_MOESM3_ESM.docx]

File name: Supplementary Data 1.

Description: List of sorted ASVs and genera after supplementation with inulin-conjugated mesoporous silica nanoparticles. Differential abundance was determined with DESeq2 (2-sided) and p values were corrected for multiple testing.

File name: Supplementary Data 2.

Description: List of active ASVs and genera after inulin supplementation. Differential abundance was determined with DESeq2 (2-sided) and p values were corrected for multiple testing.

File name: Supplementary Data 3.

Description: List of active ASVs and genera after FOS supplementation. Differential abundance was determined with DESeq2 (2-sided) and p values were corrected for multiple testing.

File name: Supplementary Data 4.

Description: List of active ASVs and genera after fructose supplementation. Differential abundance was determined with DESeq2 (2-sided) and p values were corrected for multiple testing.

File name: Supplementary Data 5.

Description: List of strains isolated with RACS after inulin supplementation and in the presence of heavy water.

File name: Supplementary Data 6.

Description: List of taxa isolated with RACS and FACS and reference type strains.

File name: Supplementary Data 7.

Description: List of active ASVs and genera after XOS supplementation. Differential abundance was determined with DESeq2 (2-sided) and p values were corrected for multiple testing.

File name: Supplementary Data 8.

Description: List of strains isolated with RACS after XOS supplementation and in the presence of heavy water.

File name: Supplementary Code 1.

Description: The script for the operation of RACS

File name: Supplementary Code 2.

Description: graphical user interface platform for running the script in Supplementary Code 1
